# Older Adult Peer Support Specialists’ Age-Related Contributions to an Integrated Medical and Psychiatric Self-Management Intervention: Qualitative Study of Text Message Exchanges

**DOI:** 10.2196/22950

**Published:** 2021-03-02

**Authors:** Mbita Mbao, Caroline Collins-Pisano, Karen Fortuna

**Affiliations:** 1 Simmons University School of Social Work Boston, MA United States; 2 Dartmouth College Lebanon, NH United States

**Keywords:** older adults, peer support, self-management, mobile technology

## Abstract

**Background:**

Middle-aged and older adults with mental health conditions have a high likelihood of experiencing comorbid physical health conditions, premature nursing home admissions, and early death compared with the general population of adults aged 50 years or above. An emerging workforce of peer support specialists aged 50 years or above or “older adult peer support specialists” is increasingly using technology to deliver peer support services to address both the mental health and physical health needs of middle-aged and older adults with a diagnosis of a serious mental illness.

**Objective:**

This exploratory qualitative study examined older adult peer support specialists’ text message exchanges with middle-aged and older adults with a diagnosis of a serious mental illness and their nonmanualized age-related contributions to a standardized integrated medical and psychiatric self-management intervention.

**Methods:**

Older adult peer support specialists exchanged text messages with middle-aged and older adults with a diagnosis of a serious mental illness as part of a 12-week standardized integrated medical and psychiatric self-management smartphone intervention. Text message exchanges between older adult peer support specialists (n=3) and people with serious mental illnesses (n=8) were examined (mean age 68.8 years, SD 4.9 years). A total of 356 text messages were sent between older adult peer support specialists and service users with a diagnosis of a serious mental illness. Older adult peer support specialists sent text messages to older participants’ smartphones between 8 AM and 10 PM on weekdays and weekends.

**Results:**

Five themes emerged from text message exchanges related to older adult peer support specialists’ age-related contributions to integrated self-management, including (1) using technology to simultaneously manage mental health and physical health issues; (2) realizing new coping skills in late life; (3) sharing roles as parents and grandparents; (4) wisdom; and (5) sharing lived experience of difficulties with normal age-related changes (emerging).

**Conclusions:**

Older adult peer support specialists’ lived experience of aging successfully with a mental health challenge may offer an age-related form of peer support that may have implications for promoting successful aging in older adults with a serious mental illness.

## Introduction 

Middle-aged and older adults with mental health conditions have a high likelihood of experiencing comorbid physical health conditions, premature nursing home admissions, and early death compared with the general population of middle-aged and older adults [1]. Despite challenges associated with mental health and comorbid physical health conditions in late life, there is a shortage of trained professionals to address the medical, psychiatric [2-4], and psychosocial age-related needs of this vulnerable population. An emerging workforce of peer support specialists aged 50 years or above is one of the fastest growing mental health workforces and may be a suitable community-based workforce to simultaneously support the mental health, physical health, and aging needs of middle-aged and older adults with a serious mental illness. A serious mental illness is defined as a diagnosable mental, behavioral, or emotional disorder that an adult has experienced in the past year that causes them serious functional impairment that substantially interferes with or limits at least one major life activity (ie, schizophrenia, bipolar disorder, and treatment refractory major depressive disorder) [5].

Older adult peer support specialists are people with a lived experience of aging into middle age and older adulthood with a mental health condition. As people with serious mental illnesses die up to 32 years earlier than the general population [1], older age is commonly defined as 50 years or above in this population of peer support specialists and their service user counterparts. For Medicaid reimbursement, older adult peer support specialists are trained and accredited by their respective state to provide support services that augment the traditional mental health system. Although accreditation and certification of older adult peer specialists varies by state, as of 2017, 41 states were billing Medicaid for peer support services [6]. Promising evidence indicates that older adult peer support specialist services reduce hospitalizations; improve engagement and treatment adherence; improve feelings of loneliness among older adults [7,8]; and promote hope, empowerment, and quality of life [9,10].

Similar to peer support specialists of any age, older adult peer support specialists employ a collaborative approach, in which caring for others creates an upward spiral of positivity to both professionals and service users [11]. This approach may be central to recovery and support bidirectional successful aging in people with serious mental illnesses and older adult peer support specialists themselves. Successful aging is defined as preventing late-life disease and disability; maintaining high cognitive, mental, and physical function; and being actively engaged in late life [12]. As such, successful aging cannot exist without the absence of disease, disability, and impairment, and thus, may not apply to middle-aged and older adults with serious mental illnesses, as the majority of this population is also diagnosed with one or more chronic health conditions [2]. As such, subjective factors of successful aging for people with serious mental illnesses may include resilience, optimism, adaptability, life satisfaction, and physical and mental health–related quality of life [12,13].

Older adult peer support specialists are increasingly using technology to deliver peer support services related to addressing *both* the mental health and physical health needs of older adults [9,10], including text messaging, videoconferencing, social media, and virtual reality. Text messages may be a low-cost high-reach intervention to support people in the community between clinical encounters with psychiatrists, social workers, etc. Clinician-supported text message exchanges have shown promising evidence of positive outcomes for improving mental health disorders and cardiac outcomes [14]. Other supportive clinician-based text message interventions have reported potential improvements in users with a comorbid diagnosis of depression and alcohol use [15]. A similar randomized controlled trial of supportive text messages for users with depression also reported positive outcomes for depression [16]. Despite evidence of clinician-based text message effectiveness for people with mental health challenges, limited literature exists on older adult peer support specialists, and unlike clinicians, their role is to intentionally disclose their lived experiences of aging with mental health challenges to support older adults. The purpose of this study was to explore older adult peer support specialists’ text message exchanges with middle-aged and older adults with a diagnosis of a serious mental illness and their nonmanualized age-related contributions to a standardized integrated medical and psychiatric self-management intervention.

## Methods

### Study Design

The study design and recruitment procedures have been described in a previously published article [17]. Briefly, a medical and psychiatric self-management intervention enhanced with the smartphone app “PeerTECH” was offered to 10 older adults with serious mental illness (ie, bipolar disorder, schizophrenia spectrum disorder, and persistent major depressive disorder) and one medical comorbidity (ie, cardiovascular disease, obesity, diabetes, chronic obstructive pulmonary disease, hypertension, and/or high cholesterol). All the participants were over the age of 60 years, and the PeerTECH intervention was delivered in the participant’s home using eModules augmented with the smartphone app (ie, it includes text messaging between older adult peer support specialists and older service users). Eight of the 10 older service users completed the intervention and were included in the current analysis. Three trained older adult peer support specialists delivered PeerTECH. All procedures were conducted in accordance with the ethical standards of the Dartmouth College Institutional Review Board and with the 1964 Helsinki Declaration and its later amendments or comparable ethical standards. Written informed consent was obtained from all participants.

An older adult peer support specialist and a research staff member met with potential participants in their homes. As part of the informed consent process, research staff members provided an overview of the study and discussed the voluntary nature of study participation and confidentiality issues. If interested, the potential participant completed written informed consent and could ask any questions as needed. This study was approved by Dartmouth College Institutional Review Board. After the informed consent form was completed by the participant, an older adult peer support specialist met with the participant in his/her home for 1 hour each week over 12 weeks.

A study of PeerTECH text messaging exchanges has been conducted with the data. The related findings have been described in detail in a previously published report [17]. Previous research explored how older adult peer support specialists used text messages to support integrated illness self-management (ie, via health behavior change, self-management therapeutic techniques, engagement in health technology, and peer support) [17]. This study seeks to expand on prior work by exploring unique older adult peer support specialists’ age-related contributions to a medical and psychiatric self-management intervention enhanced with a smartphone app.

### Older Adult Peer Support Training

Older adult peer support specialists completed their state-certified peer specialist support training. In addition to their state credentialing, as part of this study, they were provided a 4-day training by the third author (20 hours), which included the following content: (1) information about the interconnected relationship between aging, physical health, and mental health; (2) training older adults to use technology and mitigating normal age-related challenges using technology; (3) older adult mental health (eg, suicidality in late life, anxiety and depression in late life, and role of life events in mental health in older adults); (4) techniques used in PeerTECH (ie, motivational interviewing, psychoeducation, coping skills training, and behavioral tailoring for medication adherence); (5) setting personalized goals and action steps to achieve goals; (6) delivering PeerTECH sessions in person using eModules on a tablet; (7) structure of the weekly sessions; (8) using lived experience of successful aging, physical health, and mental health challenges and role play in teaching self-management skills; and (9) orientation to the smartphone app. Each older adult peer support specialist had a two to three-person caseload and worked a total of 10 hours per week, including direct care, text messaging participants, and supervision. Older adult peer support specialists were supervised by a trained older adult peer support supervisor for 1 hour each week in person or over the telephone. Verbal informed consent was obtained from older adult peer support specialists during the older adult peer support specialist training.

### Text Messaging Requirements

The smartphone intervention portion of PeerTECH was designed to reinforce in-person sessions and to provide support to people in real-world environments. Older adult peer support specialists were instructed to message participants a minimum of three times a week. There was no maximum number of text messages required. Text message exchanges were unstructured and were to focus on nonmanualized peer support, follow-up of service users’ goals, and discussions during in-person sessions facilitated by eModules (eg, “hope you are doing well on your goals- journaling and walking”). Older adult peer support specialists were encouraged to text message at times consistent with the preferences of the service users they were working with. All text message content was logged, and the time/date was recorded. Requirements for text messaging were purposely left unstructured in an effort to examine naturalistic interactions between older adult peer support specialists and service users with a diagnosis of a serious mental illness.

Smartphones and data plans were provided free of charge or service user participants could use their own smartphone. Participants were not provided incentives to send text messages; however, they were provided US $20 compensation to complete baseline, 1-month, and 3-month assessments (total of US $60 over the entire study duration). For this study, incoming and outgoing text messages were securely stored within the smartphone app database. Text message transcript data were extracted into an Excel worksheet and analyzed.

### Data Analysis

Transcripts were analyzed for eight participants and three older adult peer support specialists. The codebook consisted of a priori older adult peer support specialist and nonpeer researcher–driven codes, which were derived from text messages, and inductively derived codes from qualitative data [18]. The first and third authors read data and incorporated new codes and operational definitions from transcript coding, which is a validated approach that allows for multiple perspectives [18]. Codes were assigned to text, grouped, and checked for themes. Thematic analysis was used to summarize themes identified in the text message data [19]. Analyses assessed within-group consensus or disagreement. Member checking was employed to validate results and resolve any incongruent findings. As such, the third author contacted the participating older adult peer support specialists to discuss the key themes that emerged from the text message data. This approach helped ensure these findings are consistent with how older adult peer support specialists intend to use these text message exchanges. Quantitative data comprised the frequency of text messages by either older adult peer support specialists or service users. Frequency data were captured directly from the PeerTECH app. Frequency data were integrated at the conclusion of the study.

## Results

### Study Sample

The sample consisted of eight service user participants and three older adult peer support specialists. Service user participants had a mean age of 68.8 years (SD 4.9 years; range 62-77 years) and were primarily women (7/8, 88%), White (8/8, 100%), and married (6/8, 75%). The sample included people diagnosed with major depressive disorder (5/8, 63%), schizophrenia spectrum disorder (2/8, 25%), and bipolar disorder (1/8, 13%). Older adult peer support specialists were all aged 55 years or above. Additionally, 100% (3/3) were female, 66% (2/3) identified as White, and 33% (1/3) identified as African American.

### Text Message Exchanges

Older adult peer support specialists sent text messages to participants’ smartphones based on participants’ preferences from 8 AM to 10 PM EST on weekdays and weekends. Over the course of the 12-week intervention, a total of 356 text messages were sent. For this study, only the text message exchanges were analyzed. Five themes emerged including (1) simultaneously managing mental health and physical health issues through empowerment and technology; (2) realizing new coping skills in late life; (3) sharing roles as parents and grandparents; (4) wisdom; and (5) sharing lived experience of difficulties with normal age-related changes.

#### Using Technology to Simultaneously Manage Mental Health and Physical Health

The first and most predominant theme was using technology to simultaneously manage mental health and physical health (29/53, 55%). Older adult peer support specialists encouraged participants to take control of their mental health and physical health needs using technology. For example, an older adult peer support specialist texted as follows:

You were able to relax enough to get the exercise. It was amazing what 10 minutes of focused quiet can do. Take a timer and just do a mental relaxation exercise for 5 minutes 2x a day. It will be great for your heart. You can find guided imagery and meditations videos on YouTube also.

Another older adult peer support specialist texted as follows:

I can feel the stress you are under. In the app, there are several short videos on stress reduction like deep breathing, mindfulness, meditation that you can try to have some peace.

Another older adult peer support specialist provided tools for reducing anxiety as follows:

Doing slow deep breathing and short meditations can relax you and dissipate anxiety more effectively than cigarettes. Think about it. I will look for some YouTube videos on relaxation and/or smoking cessation.

#### Realizing New Coping Skills in Late Life

The second most predominant theme was realizing new coping skills in late life (8/53, 15%). Older adult peer support specialists purported that they are learning new skills in late life related to coping skill development. For example, an older adult peer support specialist texted as follows:

Since I have been in recovery, I am learning new skills to cope with stressful days, weeks, and months.

Another older adult peer support specialist texted as follows:

I do a lot of journaling (writing). It helps me in many ways- when I am upset; when I don't have someone to talk to and I need to get it out of my head; when I have to make a big decision...Writing has been a blessing for me especially in tough times.

#### Sharing Roles as Parents and Grandparents

The third most predominant theme was sharing roles as parents and grandparents in late life (7/53, 13%). For example, an older adult peer support specialist texted as follows:

Being (Grandma...) means so much to me. I know you love your grandchildren and in time we can work through all of this with grace and ease.

Another older adult peer support specialist texted as follows:

We as parents and women tend to put everybody first. Now it is our time to take care of ourselves.

Older adult peer support specialists used their shared experiences to help participants solve the challenges they were having with their families, which were impacting service users’ mental health. Older adult peer support specialists and older adults with mental health conditions shared similar experiences, for example, an older adult peer support specialist texted as follows:

We grandmothers can't hold back our sheer joy and love we have for our grandchildren. I wish I had a video of your “grandmother moment.” Your smile lit up the room as you shared about your excitement from hearing from your granddaughter (that's is tears of joy smile).

#### Wisdom

The fourth theme was wisdom (6/53, 11%). Wisdom, for the purpose of this manuscript, was defined as advanced-level knowledge that leads to good judgment [20]. Older adult peer support specialists and older adults with mental health conditions both offered each other wisdom regarding aging successfully. For example, one older adult peer support specialist texted as follows:

We form habits without knowing it and once we identify the “bad” habits, we can turn those unwanted habits around in 14-21 days. We just have to be persistent.

Another older adult peer support specialist texted as follows:

What you resist, persist what we focus on, we also get... as a man thinketh, so is he... What the mind can conceive and believe, it can achieve... In other words, instead of focusing on what you don't want, put your focus on what you do want. … What can you do to transform your thinking? What actions can you take to feel better today? What do you want to be focused on? WORDS HAVE POWER and so do we. We have a saying “Be, Do, Have” ex: Be Happy, Do things that make you feel happy and you will have Happiness in your life.

#### Sharing Lived Experience of Difficulties With Normal Age-Related Changes

The fifth emerging theme was sharing lived experience of difficulties with normal aging (3/53, 6%). An older adult peer support specialist texted as follows:

Sleep is a challenge for me too. It is a process; we will work on it. I added the Sleep module. I hope you can get some good tips on getting a good night sleep. I get home so late at night it’s hard for me to wind down. Let me know what works best for you, so I can try it too.

## Discussion

### Principal Findings

This exploratory qualitative study examined older adult peer support specialists’ text message exchanges with middle-aged and older adults with a diagnosis of a serious mental illness and their nonmanualized age-related contributions to a standardized integrated medical and psychiatric self-management intervention. The following themes emerged: (1) simultaneously managing mental health and physical health issues through empowerment and technology; (2) realizing new coping skills in late life; (3) sharing roles as parents and grandparents; (4) wisdom; and (5) sharing lived experience of difficulties with normal age-related changes. Older adult peer support specialists’ lived experience of aging successfully with a mental health challenge may offer an age-related form of peer support that may have implications for promoting successful aging in older adults with serious mental illnesses.

The age of older adult peer support specialists and their experiences with multimorbidity led to text message exchanges that focused on the management of mental health and physical health challenges using technologies outside of the PeerTECH platform (eg, smartphone apps, YouTube, and social media platforms). Older adults in the general population with multimorbidity are often faced with burdens of managing treatment, such as increased health care visits, refilling prescriptions, managing diet concerns, and self-managing care [14], and this is compounded by functional and structural challenges (eg, lack of transportation) associated with serious mental illnesses in late life [2]. As increasing numbers of older adults with serious mental illnesses are using technologies [21], technologies may be viable tools to support late-life self-management of mental health and physical health challenges. Further, older peer support specialists’ personal experience of coping with multimorbidity using technologies may influence service users to engage with these new technologies to support their own medical and psychiatric self-management skill development. Prior research has found that clinician [22] and peer support interactions [23] within digital mental health services facilitate engagement with technologies. As such, older adult peer support specialists’ shared experience may have a role in influencing engagement in digital interventions among older adults with serious mental illnesses.

The shared lived experience of parenting and grandparenting between older adult peer support specialists and service users may have facilitated the development of a supportive alliance. Older adult peer support specialists’ practice principles, unlike those of clinicians, encourage sharing of their lived experiences (or self-disclose) to support the recovery of individuals. The use of intentional self-disclosure may have facilitated the development of a supported alliance between older adult peer support specialists and older adult service users.

Through sharing of experiences, older adult peer support specialists offered wisdom related to navigating some of the challenges resulting from age, illness, and life experiences.

Wisdom is a prototype of successful aging [24] and has been found to enhance mental health and promote well-being in older adults (without serious mental illnesses) [25]. Possibly, through bidirectional sharing of knowledge of aging, illness, and life experiences, both older adult peer support specialists and older service users may assist one another in navigating some of the challenges of aging successfully with a serious mental illness, which could support older adults between clinical encounters. This is also an important area for future inquiry. As peer support is a nonmanualized form of support [26], peer support specialists aged 50 years or above may offer different lived experience expertise than their younger adult peer support specialist counterparts, based on their level of expert knowledge (ie, wisdom).

This study is not without limitations. First, it is not known whether we met saturation. Qualitative interviews are conducted until there is saturation of data (ie, saturation means that researchers reach a point in their analysis where sampling more data will not lead to more information related to their research questions) [27]. By drawing from grounded theory design [18], saturation would generally occur with 20 to 30 participants in total [28]; however, the sample size was small because the primary study was conducted to assess feasibility [9]. It is important to note that findings cannot be generalized; however, the themes identified can be used to guide the development of peer support text-messaging services as an adjunct to evidence-based interventions [9]. Further, we were unable to stratify our data by demographic characteristics owing to the sample size. For example, one peer had a master’s degree in social work, and this advanced educational background likely influenced the person’s delivery of services. Second, peers met in person with participants over a 12-week time frame, and in-person follow-up discussions from text messages are not reported. Third, the sample involved a heterogeneous group of people with psychotic disorders and mood disorders that predominately included those with major depressive disorder. Fourth, the participants in this study were all receiving mental health services, and therefore, our findings cannot be generalized to individuals with serious mental illnesses not enrolled in care or without access to mental health services. Finally, the results elucidate text message themes between older adult peer support specialists and older adults with serious mental illnesses and chronic health conditions; however, it is not known whether the peer-to-participant text message exchanges can improve self-management and other clinical outcomes.

### Conclusion

Older adult peer support specialists are an emerging part of the service delivery system for older adults. Older adult peer support specialists offered text message–based age-related experiential contributions to support aging successfully with a mental health and physical health condition. Through older adult peer support specialists’ wisdom, sharing of new late-life coping skills and similar age-specific roles in life and encouragement to use technology to support medical and psychiatric self-management may promote engagement in nontraditional support services (ie, YouTube) and support for older adults with serious mental illnesses in the community between clinical encounters.

## References

[1] Walker E, McGee RE, Druss BG (2015). Mortality in mental disorders and global disease burden implications: a systematic review and meta-analysis. JAMA Psychiatry.

[2] Bartels S, DiMilia PR, Fortuna KL, Naslund JA (2018). Integrated Care for Older Adults with Serious Mental Illness and Medical Comorbidity: Evidence-Based Models and Future Research Directions. Psychiatr Clin North Am.

[3] Everett A (2019). Bringing Awareness to the Mental Health of Older Adults. SAMHSA.

[4] Eden J, Maslow K, Le M, Blazer D (2012). The Mental Health and Substance Use Workforce for Older Adults. In Whose Hands? Assessing the Service Needs of Older Adults with Mental Health and Substance Use Conditions. The Mental Health and Substance Use Workforce for Older Adults. In Whose Hands? Assessing the Service Needs of Older Adults with Mental Health and Substance Use Conditions.

[5] (2020). Adults With SMI and Children/Youth With SED. SAMHSA.

[6] Videka L, Neale J, Page C, Buche J, Beck AJ, Wayment C, Gaiser M (2019). National analysis of peer support providers: Practice settings, requirements, roles, and reimbursement. Behavioral Health Workforce.

[7] Conner K, Cadet T, Brown M, Barnett J (2018). The Impact of Peer Support on the Risk of Future Hospital Readmissions among Older Adults with a Medical Illness and Co-Occurring Depression. Social Sciences.

[8] Gagne C, Finch WL, Myrick KJ, Davis LM (2018). Peer Workers in the Behavioral and Integrated Health Workforce: Opportunities and Future Directions. Am J Prev Med.

[9] Fortuna K, Lohman MC, Gill LE, Bruce ML, Bartels SJ (2017). Adapting a Psychosocial Intervention for Smartphone Delivery to Middle-Aged and Older Adults with Serious Mental Illness. Am J Geriatr Psychiatry.

[10] Fortuna KL, Naslund JA, LaCroix JM, Bianco CL, Brooks JM, Zisman-Ilani Y, Muralidharan A, Deegan P (2020). Digital Peer Support Mental Health Interventions for People With a Lived Experience of a Serious Mental Illness: Systematic Review. JMIR Ment Health.

[11] Feeney B, Collins NL (2015). A new look at social support: a theoretical perspective on thriving through relationships. Pers Soc Psychol Rev.

[12] Jeste DV, Ardelt M, Blazer D, Kraemer HC, Vaillant G, Meeks TW (2010). Expert consensus on characteristics of wisdom: a Delphi method study. Gerontologist.

[13] Ibrahim F, Cohen CI, Ramirez PM (2010). Successful Aging in Older Adults With Schizophrenia: Prevalence and Associated Factors. The American Journal of Geriatric Psychiatry.

[14] Hoffmann T, Jansen J, Glasziou P (2018). The importance and challenges of shared decision making in older people with multimorbidity. PLoS Med.

[15] Agyapong V, Ahern S, McLoughlin DM, Farren CK (2012). Supportive text messaging for depression and comorbid alcohol use disorder: single-blind randomised trial. J Affect Disord.

[16] Agyapong V, Juhás M, Ohinmaa A, Omeje J, Mrklas K, Suen VYM, Dursun SM, Greenshaw AJ (2017). Randomized controlled pilot trial of supportive text messages for patients with depression. BMC Psychiatry.

[17] Fortuna K, Naslund JA, Aschbrenner KA, Lohman MC, Storm M, Batsis JA, Bartels SJ (2019). Text message exchanges between older adults with serious mental illness and older certified peer specialists in a smartphone-supported self-management intervention. Psychiatr Rehabil J.

[18] Martin P, Turner BA (2016). Grounded Theory and Organizational Research. The Journal of Applied Behavioral Science.

[19] Braun V, Clarke V (2006). Using thematic analysis in psychology. Qualitative Research in Psychology.

[20] Baltes PB, Kunzmann U (2003). Wisdom. The Psychologist.

[21] Ben-Zeev D, Davis KE, Kaiser S, Krzsos I, Drake RE (2013). Mobile technologies among people with serious mental illness: opportunities for future services. Adm Policy Ment Health.

[22] Mohr D, Cuijpers P, Lehman K (2011). Supportive accountability: a model for providing human support to enhance adherence to eHealth interventions. J Med Internet Res.

[23] Fortuna KL, Brooks JM, Umucu E, Walker R, Chow PI (2019). Peer Support: a Human Factor to Enhance Engagement in Digital Health Behavior Change Interventions. J. technol. behav. sci.

[24] Parisi J, Rebok GW, Carlson MC, Fried LP, Seeman TE, Tan EJ, Tanner EK, Piferi RL (2009). Can the Wisdom of Aging be Activated and Make a Difference Societally?. Educ Gerontol.

[25] Jeste D, Lee EE (2019). The Emerging Empirical Science of Wisdom: Definition, Measurement, Neurobiology, Longevity, and Interventions. Harv Rev Psychiatry.

[26] Solomon P (2004). Peer support/peer provided services underlying processes, benefits, and critical ingredients. Psychiatr Rehabil J.

[27] Seale C, Gobo G, Gubrium JF, Silverman D (2004). Qualitative Research Practice.

[28] Creswell JW (2007). Qualitative Inquiry and Research Design: Choosing Among Five Approaches.

